# Metabolome Profiling: A Breeding Prediction Tool for Legume Performance under Biotic Stress Conditions

**DOI:** 10.3390/plants11131756

**Published:** 2022-07-01

**Authors:** Penny Makhumbila, Molemi Rauwane, Hangwani Muedi, Sandiswa Figlan

**Affiliations:** 1 Department of Agriculture and Animal Health, School of Agriculture and Life Sciences, College of Agriculture and Environmental Sciences, University of South Africa, 28 Pioneer Ave, Florida Park, Roodeport 1709, South Africa; rauwaneme@gmail.com (M.R.); figlas@unisa.ac.za (S.F.); 2 Research Support Services, North West Provincial Department of Agriculture and Rural Development, 114 Chris Hani Street, Potchefstroom 2531, South Africa; hmuedi@nwpg.gov.za

**Keywords:** legumes, metabolomics, biotic stress, stress tolerance, metabolome annotation

## Abstract

Legume crops such as common bean, pea, alfalfa, cowpea, peanut, soybean and others contribute significantly to the diet of both humans and animals. They are also important in the improvement of cropping systems that employ rotation and fix atmospheric nitrogen. Biotic stresses hinder the production of leguminous crops, significantly limiting their yield potential. There is a need to understand the molecular and biochemical mechanisms involved in the response of these crops to biotic stressors. Simultaneous expressions of a number of genes responsible for specific traits of interest in legumes under biotic stress conditions have been reported, often with the functions of the identified genes unknown. Metabolomics can, therefore, be a complementary tool to understand the pathways involved in biotic stress response in legumes. Reports on legume metabolomic studies in response to biotic stress have paved the way in understanding stress-signalling pathways. This review provides a progress update on metabolomic studies of legumes in response to different biotic stresses. Metabolome annotation and data analysis platforms are discussed together with future prospects. The integration of metabolomics with other “omics” tools in breeding programmes can aid greatly in ensuring food security through the production of stress tolerant cultivars.

## 1. Introduction

Leguminous crops such as *Arachis hypogaea* (groundnut), *Glycine max* (soybean), *Phaseolus vulgaris* (common bean), *Pisum sativum* (common pea), *Cicier arietinum* (chickpea), *Vigna anguiculata* (cowpea), *Vicia faba* (faba bean), *Lens culinaris* (lentil), *Cajanus cajan* (pigeon pea), *Lupinus* spp. (lupin), and *Vigna subterranean* (bambara bean) contribute to the improvement of ecosystems [1,2,3], nutrition and food security [4,5,6,7]. Although legumes contribute greatly to food security, their production globally is hindered by biotic stresses that include nematodes, viruses, insect pests, and bacterial and fungal pathogens [8,9,10]. The occurrence of biotic stresses in legume production systems has impacted negatively on production and has resulted in significant yield losses globally [11,12,13]. In many breeding programmes, the key objective is to develop crop varieties that are adaptable to an array of stressors in order to meet global food demands [14,15,16], thus addressing sustainable development goals 1 and 2 of the United Nations [17]. Legume programmes have been improving gradually over the years and have advanced from traditional methods of breeding to using genomic tools [18]. Traditional breeding techniques rely mostly on manual selection and the crossing of genotypes with desirable traits, and although these methods have contributed greatly to legume breeding, the genetic gain was often not statistically significant [19].

Contemporary biotechnology tools including next generation sequencing (NGS) platforms have aided many breeding programmes with provision of genetic data that traditional breeding techniques cannot fully reveal [20]. Biotechnological “omics” approaches have contributed greatly to breeding aimed at the improvement of plant stress tolerance by providing insight into genetic diversity, genotype variations, genetic maps and other useful information pertaining to the genetics of plant populations [21,22]. Despite the importance of genomic data generated by the other omics platforms (transcriptomics, transgenomics, epigenomics), plants produce molecular compounds with molecular weights expressed in abundance and are responsible for biochemical functions under different environments [23]. Metabolomics highlights metabolite expressions and changes, together with their interactions and phenotypic characters of plants under stress conditions. When plants are exposed to stress, metabolic homeostasis alterations occur, requiring the plant to adjust its metabolomic pathways, and this phenomenon is referred to as acclimation [24,25,26,27]. When this process occurs, the plant activates signal transduction pathways that set off the assembly of proteins and metabolomic compounds that aid in reaching a new homeostasis [28,29]. Furthermore, metabolome analysis provides information on the metabolomic pathways that are responsible for complex processes that occur when a plant is exposed to stress conditions [20].

A detailed review of metabolomic studies focused on specific biotic stressors of legumes can aid in identifying gaps and create an interactive platform for researchers to conduct, and possibly collaborate on, more studies aimed at improving legume production in the world. This is because the dimensionality of large data sets generated through metabolomics can be interpreted holistically utilising multivariate data analysis [30]. This will further highlight the importance of metabolite detection in breeding programmes and techniques that can be employed for different objectives since metabolites relate to phenotypic and genomic data [9]. This review reports on metabolomics as a breeding prediction tool in legume breeding under biotic stress. We also briefly discuss the impact of metabolomics in legume breeding programmes aimed at improving biotic stress tolerance.

## 2. Biotic Stressors of Legumes

### 2.1. Insect Pests

Insect pests attack legume crops by boring, webbing and damaging plant parts such as the leaves, pods, stems and roots [31,32]. In addition to attacking plants, insect pests may also act as vectors for pathogens that negatively impact crop production systems [33]. Insect pests such as aphids [33,34], pod borers [31,35], thrips [36,37] and whiteflies [38,39] have been reported to feed on legume crops, among others. The use of biological enemies of pests, cultural control (crop rotation, mulching, intercropping, etc.), mechanical control (water hosing at high pressure), chemical application and integrated pest management strategies have been recommended for the control of insect pests in legumes [39,40,41,42]. These efforts have been found to be effective in reducing insect severity in legumes [39,43]. However, the insects are constantly adapting to control measures used in production systems [44]. Breeding for tolerance to insect pests is the most sustainable approach and this requires an understanding of the plant’s signal pathways that respond to insect attack [45].

Pathways expressed in rice infested with caterpillars included flavonoids, phenolic acids, amino acids and derivatives. These improved the production of cytosolic calcium ions that signal herbivore attack to the plant [46]. Maize infested with *Monolepta hieroglyphica* revealed significant up-/down-regulation of metabolites derived from sugar and amino acid pathways that might be responsible for resistance. Similar results were reported in cabbage infested with aphids [47]. Insect–plant metabolomic response of leguminous crops has been conducted for red clover, pea and alfalfa in a composite study with aphid infestation. Triterpene, flavonoid and saponin enriched pathways were found to be responsive to aphid attack [34]. Flavonoids and amino acids have also been found to be significantly enriched in alfalfa infested with thrips [48]. However, limited studies have been conducted on the host-plant metabolomic response of leguminous crops to insects, as well as to other biotic stressors. These studies could have far-reaching impacts on stress biomarker identification with potential benefits in legume improvement programmes.

### 2.2. Diseases of Legumes

#### 2.2.1. Bacterial Diseases

Bacterial diseases of legumes can be categorised into leaf blights, leaf spots/bacterial wilts and other multiple symptoms of sprout rot and dwarfism [49]. Their symptoms are based on the tissues that they infiltrate (leaves, stems and roots) [50]. Legume bacterial diseases are known to cause yield losses of up to 50%, which negatively impacts economic gains and food security [51]. The two plant bacterial pathogens *Xanthomonas axonopodis* and *Pseudomonas syringae* are known worldwide for causing bacterial blight [49,52]. Symptoms of infection usually occur on all aerial parts of the plant, and in severe incidences, defoliation and wilting occur [52,53]. Like bacterial blight, another disease that threatens legume production is bacterial wilt, caused by *Curtobacterium flaccumfaciens pv. Flaccumfaciens* [54]. The pathogen has created new variants that cause damage to legume crops worldwide by causing leaf chlorosis in plants. In fields where the disease occurs, upon plant maturation and shattering of seeds, the infected seed replants itself and allows the pathogen to thrive from generation to generation [54,55]. The control of bacterial diseases has relied on integrated approaches that limit the survival of pathogens. This includes crop rotation and the use of pathogen free certified seed [52]. These measures are only effective to a limited extent, and detecting pathogens in seed is not an easy task for farmers. A promising and more long-term method for the control of bacterial diseases would be the utilisation/breeding of tolerant varieties [56,57].

The evaluation of metabolite profiles in citrus infected with huanlongbing caused by the bacterium ‘*Candidatus Liberibacter asiaticus*’ reported distinct sugars as well as amino and organic acids expressed in the roots, thus giving insight on resistance [58]. Metabolomic compounds synthesized from flavonoids, amino and phenolic acids act as protective agents in the xylem of oat plants when infected with halo blights caused by *P. syringae pv.* by repairing the cell wall [59]. Similar metabolomic pathways including phenols and acetates have been reported in tomato infected with bacterial wilt caused by *Ralstonia solanacearum* [60]. To date, there is little to no information from metabolomic studies on the response of leguminous crops to bacterial disease infection to aid breeders with biomarker discovery.

#### 2.2.2. Fungal Diseases

The occurrence of fungal diseases in legume production areas is known to cause substantial yield losses of up to 100% [59]. Fungal pathogens can cause infection at any plant growth stage (emergence, seedling, vegetative and reproductive stage) by attacking organs and tissues that are involved in the transportation of water and nutrients [61,62]. Upon infection, these pathogens degrade the plant cell wall, which consequently results in the death of the plant, especially if the variety grown does not have any resistant genes [63]. Root rot caused by *Rhizoctonia solani*, *Fusarium solani*, *Fusarium oxysporum* and *Aphanomyces euteiches* and fungal wilt caused by *Formae speciales* are some of the most destructive fungal diseases that limit the productivity of legume crops worldwide [64]. The pathogen *R. solani* is considered one of the most destructive fungal pathogens that usually infects the roots and hypocotyl of the plant through penetration of the appressoria [63]. At pre-emergence and post-emergence plant growth stages, *R. solani* causes symptoms of damping-off, root rot and stem canker [65]. Under greenhouse conditions, the seedling survival of some leguminous crops may be less than 5% [66]. The pathogen may further infect the plant’s fruits in highly humid conditions, thus reducing crop quality and yield [67]. *Fusarium* spp. are also predominant pathogens that interfere with plant growth by causing damping off and root rot [68]. In African small-scale farms, yield losses of up to 100% caused by the *F. solani* pathogen in common bean have been reported [69]. In addition, *A. euteiches* is a soil-borne fungal pathogen that poses a threat to legume production by causing wilting, root rot and consequently yield losses of up to 80% [70,71].

The management of fungal diseases is problematic due to the complexity of these pathogens [72]. Over the years, management has been implemented by integrating conventional methods such as crop rotations, increased greenhouse temperatures, biological enemies and chemical use [73]. The use of fungicides has been a promising avenue for the control of fungal pathogens. However, chemicals used to control pathogens have an immense economic and environmental impact [74]. This has led to the exploration of using biological control measures such as bacterium and fungal strains as environmentally friendly alternatives to control pathogens that attack plants [75]. *Trichoderma* spp. are widely used strains for the biological control of fungal diseases. Beneficial strains of *T. velutinum* have been found to be an effective biological control measure that promotes the accumulation of metabolites that are responsible for defence in common bean infected with *F. solani*. Even though numerous strains have been found to be effective in controlling fungal diseases, legislation in many countries regarding the use of biopesticides and their shelf life is still a challenge [76,77]. The development of disease-resistant cultivars using genomic technologies can aid in improving legume productivity worldwide [54]. Legume metabolomics focussed on breeding for disease resistance can be beneficial to breeding programmes by increasing the availability of resistant genotypes that are released to farmers [78].

The metabolomic profiling of leguminous crops has been conducted in common bean and provided major findings in relation to metabolomic pathways including amino acids, flavonoids, isoflavanoids, purines and proline metabolism, which were shown to promote plants’ potential for defence against *Fusarium* pathogens [79]. In addition, Mayo-Prieto et al. [80] also reported amino acids, peptides, carbohydrates, flavonoids, lipids, phenols, terpenes and glycosides that were up-/down-regulated as a defence mechanism by the common bean plant against the pathogen *R. solani*. Similar results have been reported in other leguminous crops including chickpea infected with *F. oxysporum*, soybean infected with *Aspergillus oryzae/Rhizopus oligosporus*, pea infected with *Dydymella pinodes* and *R. solani* (Table 1) [81,82]. Intensifying the fungal–legume metabolomic research worldwide will aid in understanding the biochemical properties of these leguminous crops in response to disease stress.

#### 2.2.3. Viral Diseases

Viral pathogens attack many crops, including legumes, by causing the yellowing of leaves, stunting and poor pod setting, which result in poor yields [65]. Major viral diseases causing production losses in legumes belong to the *Nanoviridae*, *Luteovridae* and *Poltyvridae* families. These diseases cause the necrosis of plants, and their identification requires molecular techniques. Over the years, the accurate identification of viruses has improved because of an increasing number of available genomic platforms. [49,66]. Viruses attach themselves to specific sites of vectors such as insects (aphids, beetles, etc.) and remain there until transmission to their host occurs [67]. The control of viral diseases is difficult and thus requires adherence to quarantine prescripts, removal of inoculum sources, adjustments of planting dates, intercropping, crop rotation, chemical application aimed at controlling pests (elimination of vectors) and the use of tolerant/resistant genotypes [68].

Utilising metabolomic techniques on the *Citrus tristeza* virus of Mexican lime *Citrus aurantifolia* revealed up-/down-regulation of amino acids, alkaloids and phenols during infection, thus signalling pathogen defence when different strains of the virus were utilised [83]. In stems of *Amarathus hypochondriacus* L. infected with *Ageratum enation* virus, alkaloids, amino acids, dicarboxylic acids, glutamine and sugars may increase or decrease in concentration as a mechanism to improve overall respiratory metabolism [84]. Studies on the response of leguminous crops to viral disease infection are limited, thus requiring more research in order to fully understand the underlying information relating to metabolites expressed under virus pressure.

### 2.3. Parasitic Weeds

Unlike “normal” weeds that disadvantage the plant greatly, parasitic weeds on the other hand extensively extract moisture, nutrients, photosynthates and other resources from the host plant [69]. When parasitic weeds are not controlled, the extraction of resources continues, consequently extinguishing the crop [70]. Roomrape species, *Striga gesnerioides* and *Alectra vogelii* are problematic parasitic weeds that cause yield losses in many legume production areas in Sub-Saharan Africa [71]. Biological control [69], intercropping [72], chemical application and cultural practices (timely planting) are recommended for the control of parasitic weeds [73]. However, these are often not successful, and the fight against parasitic weeds lies within breeding for resistance [71,73]. Although breeding for resistance will aid in controlling parasitic weeds, the complexity and low heritability is a challenge that breeders face when breeding for parasitic weed resistance [71,73,74]. Initiatives to use breeding prediction tools such as metabolomic techniques for parasitic weed resistance have been explored in rice to study and dissect *S. hermonthica* resistance [85]. This study reported the phenylpropanoid pathway, which contributes to the formation of lignin in rice, to be an important pathway that can be utilised for resistance to *S. hermonthica*. There is a deficit on metabolomic experiments that evaluate the performance of legumes under parasitic weed conditions.

### 2.4. Parasitic Nematodes

Legumes are famous for their ability to fix nitrogen by using rhizobium, which is a mutualist bacterium [75]. However, the presence of parasitic nematodes reduces rhizobia activity, which leads to poor nodulation [76]. Parasitic nematodes invade the roots of plants and form an indefinite feeding area, which, in turn, can affect root development, thus leading to poor plant growth [77]. *Heterodera* and *Globodera* spp. are root knot and cyst nematodes that affect many crops including legumes, resulting in over 12% yield losses [78]. The presence of parasitic nematodes often leads to infection by other pathogens including *fusarium* spp.; therefore, the utilisation of sustainable control strategies for other pathogens is essential for legumes [74]. Soybean evaluated under *Melodegyne pinodes* and *Heterodera glycines* pressure exhibited phenylpropanoids, cysteine, methionine, alkaloid and tropane pathways that can be attributed to resistance properties of the crop to nematodes [86]. The in-depth exploration of metabolites of other crops including legumes would be beneficial to understanding nematode–crop biological interactions.

**Table 1:** Summary of metabolomic studies conducted in response to biotic stress in leguminous crops using different platforms such as GC-MS, LC-QqQ-MS, LC-MS, LC-obitrap-MS, UHPLC-MS, ^1^H NMR and GC-MS/TOF.

Legume	Biotic Stress	Classification	Method	Total Metabolites	Reference
*C. arietinum*	*Fusarium oxysporum*	Fungal	GC-MS	72	[87]
*G. max*	*Aspergillus oryzae/Rhizopus oligosporus*	Fungal	LC-QqQ-MS	489	[88]
	*Heterodera glycines*	Nematode	GC-MS	20	[86]
*M. sativa*	*Thysanoptera* spp.	Insect	LC-MS	772	[48]
	*Acyrthosiphon pisum Harris*	Insect	LC-Obitrap-MS/UHPLC-MS	107	[34]
*P. sativum*	*Acyrthosiphon pisum Harris*	Insect	LC-Obitrap-MS/UHPLC-MS	57	[34]
	*Didymella pinodes*	Fungal	LC-MS/MS	31	[89]
	*Rhizoctonia solani*	Fungal	^1^H NMR	126	[81]
	*Didymella pinodes*	Fungal	GC-MS/TOF	39	[82]
*P. vulgaris*	*Fusarium solani*	Fungal	UPLC	743	[79]
	*Trichoderma velutinum/Rhizoctotonia solani*	Fungal	LC-MS	216	[80]
*T. pratense*	*Acyrthosiphon pisum Harris*	Insect	LC-Obitrap-MS/UHPLC-MS	103	[34]
*V. faba*	*Acyrthosiphon pisum Harris*	Insect	LC-Obitrap-MS/UHPLC-MS	13	[34]

## 3. Legume Metabolomics

### 3.1. Metabolome Profiling Techniques

**Figure 1:**
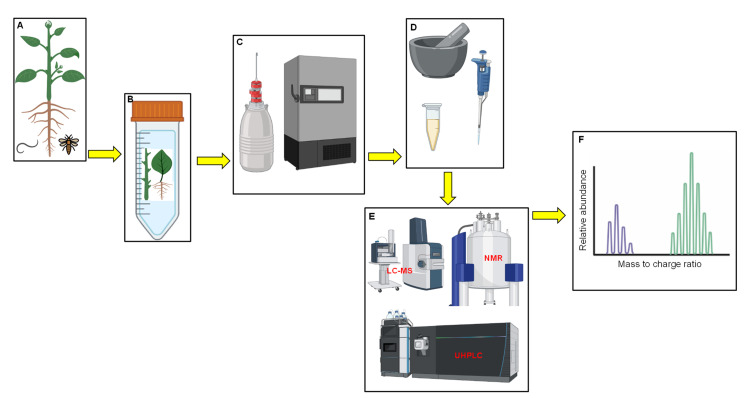
Flow diagram summarizing steps taken for metabolomic sample analysis in biotic stress experiments. Plant under biotic stress (**A**), samples from selected plant parts in a tube (**B**), snap freezing samples in liquid nitrogen and later stored in an ultra-freezer (**C**), extraction of metabolites in accordance with recommended protocols (**D**), metabolome analysis technologies (E), generation of raw spectral data (**F**).

The use of metabolomics has been applauded for its ability to provide detailed prospects by in-depth study of crop biology. Information that is derived from metabolomic tools can be translated to assess phenotypic changes/biomarkers, gene changes and, also, to distinctively support other genomic experiments [79,80]. Furthermore, metabolomic studies can be applied for polygenic traits and prediction of epistatic effects [79,88]. The overall success of detecting metabolites and their changes depends on utilising analytical techniques that can detect compound concentrations, proportions and molecular weights [81,82,89]. The concept of metabolome profiling was introduced with the use of mass spectrometry and at a later stage, gas chromatography was also introduced [87]. Since the inception of the latter, metabolome profiling using both spectrometric and chromatographic techniques have been improving [30,90]. Different strategies are utilised for compound profiling in metabolomics, including metabolite profiling, fingerprinting and target analysis [91,92]. Metabolite fingerprinting compares “fingerprints” of metabolites [93]. The profiling analyses broader groups of metabolites that are related to specific pathways or compound classes, while target analysis is utilised for targeting specific metabolic pathways and observes the occurrences of modifications [94]. Protocols for both metabolite profiling and fingerprinting in stress experiments involve the sample acquisition from a stressed plant (leaves, stems or roots; Figure 1A) that are cut and placed in a labelled tube (Figure 1B). Dewar with liquid nitrogen is ideal for snap freezing samples in the field and a laboratory ultra-freezer with a temperature above −60 °C is recommended for sample preservation to avoid dehydration (Figure 1C). The stored samples are then crushed, and extraction is conducted in preparation for metabolite analysis, using the appropriate technology that generates spectral data (Figure 1D–F).

### 3.2. Metabolite Profiling

Metabolite profiling is important in studying organisms’ biochemical pathways [88]. Numerous technologies such as gas chromatography-mass spectrometry (GC-MS), liquid chromatography-mass spectrometry (LC-MS), nuclear magnetic resonance (NMR), capillary electrophoresis-MS (CE-MS) and Fourier transform-infrared (FT-IR) spectroscopy are commonly used analytical platforms for metabolite profiling [49,95]. The unique properties of these profiling techniques together with their applications, limitations and successes in plant metabolomics have been discussed by numerous researchers [30,96,97,98,99]. There are limited studies on the metabolome profiling of legume crops evaluated under insect stress. Although not a model for legume crops, metabolomic profiling has been performed on *Medicago sativa* (a close relative of the model legume crop *M. truncatula*) under insect stress (Table 1) [34,48]. In plant–insect interactions, a metabolome profiling study on alfalfa cultivars reported the production of numerous up-regulated metabolites in response to infestation by thrips using LC-MS (Table 1). Among the metabolite classes were amino acids together with derivatives that produced toxic amino acids released by the plant in response to insect attack [48]. Similar metabolites analysed using UHPLC-MS were also reported for pea (*P. sativum*), red clover (*Trifolium pratense*) and other alfalfa genotypes in response to biotic stress [34]. In addition, Narula et al. [87] reported a large number of metabolites that were up-regulated and down-regulated when chickpea was infected with *F. oxysporum* using GC-MS as a metabolome profiling tool. Similar results were also reported for common bean infected with *F. solani* [79], *T. velutinum* and *R. solani* [80] (Table 1). Among the primary metabolites reported, amino acids, alcohols and alkaloids were upregulated. Precursor molecules of these metabolites were found to be responsible for defence and energy provision for the plant [91]. More studies have been reported on *P. sativum* focusing on metabolite profiling under biotic stress (Table 1), particularly fungal pathogens [92,100,101]. For example, using ^1^H NMR, young pea plants showed a heightened production of amino acids that signal the production of the metabolite proline during fungal infection [81]. However, as the plant grows older, its energy requirements change, and proline production reduces. Overall, the down-regulation of metabolites can be used as a guideline for selecting resistant/tolerant varieties. Varieties resistant to pathogens also produce sulphur as a defence strategy. Resistant cultivars tend to have increased sulphur assimilation with high energy accumulation from sugar metabolites (nitrogen mobilization) for restoration of damaged plant cells [92].

## 4. Metabolome Data Processing and Annotation Tools Used in Legume Stress Tolerance

**Figure 2:**
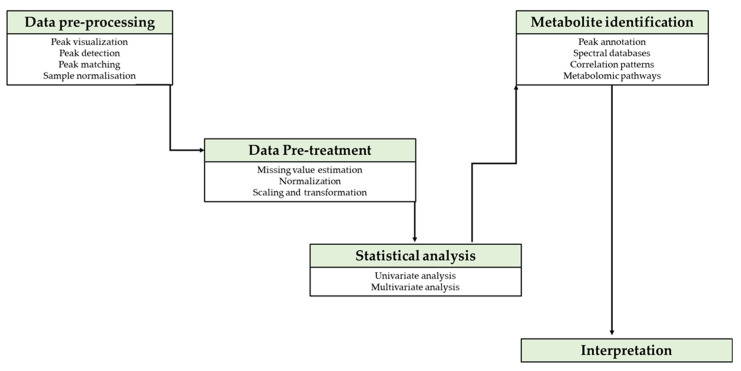
Flow diagram illustrating data handling steps for metabolomic experiments. After acquiring raw data, pre-processing, pre-treatment and statistical analysis are required prior to interpretation of results.

Metabolome usage has grown rapidly because of its provision of the cellular function data of small molecules (<1500 Da) linked to more than 40,000 metabolites that are registered on numerous databases [102]. Data generated by metabolomic technologies such as GC-MS, LC-MS and NMR, amongst others, are enormous and require software tools that are able to visualise, detect peaks, normalize/transform the sample data, annotate, identify, quantify and statistically analyse targeted/untargeted metabolite variations, in accordance with applied algorithms for univariate/multivariate analysis (Figure 2) [103,104]. There is no single tool that can unravel information from a metabolome profile; thus, analysis integrates numerous databases and requires algorithms that are provided by an array of tools [105]. Studies of metabolites in crops use an array of statistical platforms to evaluate variations of metabolites in different stress environment [106]. In legumes, metabolome data processing platforms (Table 2) used in studies of biotic stress for legumes include R and SIMCA [48,81]. Software such as SIMCA, Analyst software, STAT GRAPHICS Centurion, Labsolutions, ChromaTOF and agilent software MassHunter require licensing for metabolome data processing. However, there are numerous web-based accessible platforms that can be used for data processing, metabolome annotation and visualisation such as R, XCMS, MetaboAnalyst, METLIN, KEGG, HMBD, MeV, MetLAB and others (Table 2 and Table 3) [103].

The representation of biological networks is important in metabolomics, as it gives representation of relationships or patterns that occur in metabolomic pathways. There are numerous metabolomic pathway databases that aid in grouping metabolites with similar functions. Metabolomic pathway databases including KEGG, cytoscape, MapMan and iPath, among others, are applicable to plants [103,107].

## 5. Conclusions

Legume crops are grown in most regions of the world because they provide food security for many households. With the current climate crisis, the production of crops that are adaptable to biotic and abiotic stress is paramount. Legumes are produced in semi-arid environments and in these production areas, multiple stressors are prevalent. Plant stress response is a very complex phenomenon that researchers are constantly striving to understand by making use of high-throughput techniques. The integration and application of omics tools in agriculture has evolved and broadened the understanding of the underlying biochemical and molecular mechanisms of crops grown in diverse environments. Metabolomic studies are already becoming one of the omics tools used for breeding strategies. However, strong bioinformatics skills are needed for the processing and manipulation of the data. Furthermore, metabolomic database availability should be improved in order to accelerate information availability for legume crops. Additionally, studies that integrate metabolomics with other omics tools should aim to elaborate on the metabolomic aspects. For example, in many studies integrating transcriptomics and metabolomics, the information tends to be denser for gene expression than for metabolomics. In such cases, metabolome specific papers should be published separately to avoid complexity of integrating all the data and suppressing metabolomic information. Overall, the integration of metabolomics with other omics tools provides a powerful strategy to unravel plant–pest/pathogen interaction in biotic stress environments.

## Figures and Tables

**Table 2:** Statistical tools and databases used for metabolome data processing and annotation in legume biotic stress studies.

Legume	Statistical Tool/Database Name	Access Domain (URL, Accessed on 28 April 2022)	Function	Reference
*P. vulgaris*	Analyst software	https://sciex.com/products/software/analyst-software	Data processing Metabolite annotation	[79]
	R	https://www.r-project.org/	Data processing	
	KEGG	https://www.genome.jp/kegg/kegg2.html	Metabolomic pathways	
	Agilent MassHunter	https://www.agilent.com/en/promotions/masshunter-mass-spec	Data processing	[80]
	Pubchem	https://pubchem.ncbi.nlm.nih.gov/	Metabolite annotation	
	HMBD	https://hmdb.ca/	Metabolite annotation	
	CAS	https://www.cas.org/	Metabolite annotation	
	ChemSpider	http://www.chemspider.com/	Metabolite annotation	
	METLIN	https://metlin.scripps.edu/landing_page.php?pgcontent=mainPage	Metabolite annotation	
*M. sativa*	Analyst software	https://sciex.com/products/software/analyst-software	Data processing Metabolite annotation	[48]
	R	https://www.r-project.org/	Data processing	
	KEGG	https://www.genome.jp/kegg/kegg2.html	Metabolomic pathway analysis	
	XCMS	https://xcmsonline.scripps.edu/landing_page.php?pgcontent=institute	Data processing	[34]
	MetaboAnalyst	https://www.metaboanalyst.ca/	Data processing	
	R	https://www.r-project.org/	Data processing	
	METLIN	https://metlin.scripps.edu/landing_page.php?pgcontent=mainPage	Metabolite annotation	
	MassBank	https://massbank.eu/MassBank/	Metabolite annotation	
	HMBD	https://hmdb.ca/	Metabolite annotation	
	LipidMaps	https://www.lipidmaps.org/	Metabolite annotation	
	KEGG	https://www.genome.jp/kegg/kegg2.html	Metabolomic pathways	
	Labsolutions	https://www.shimadzu.com/an/products/software-informatics/software-option/labsolutions-cs/index.html	Data Processing Metabolite annotation	
*P. sativum*	COVAIN toolbox	https://bio.tools/covain	Data processing Metabolite annotation	[89]
	STATGRAPHICS Centurion	https://www.statgraphics.com/	Data processing	
	R Studio	https://www.rstudio.com/	Data processing	
	ChromaTOF	https://www.leco.com/product/chromatof-software	Data processing and Metabolite annotation	[82]
	SIMCA	https://www.sartorius.com/en/products/process-analytical-technology/data-analytics-software/mvda-software/simca	Data processing and Metabolite annotation	
	JMP software	https://www.jmp.com/support/downloads/JMPG101_documentation/Content/JMPGUserGuide/IN_G_0018.htm	Data processing and Metabolite annotation	[81]
	SIMCA	https://www.sartorius.com/en/products/process-analytical-technology/data-analytics-software/mvda-software/simca	Data processing and Metabolite annotation	
	R	https://www.r-project.org/	Data processing	
	KEGG	https://www.genome.jp/kegg/kegg2.html	Metabolomic pathway analysis	
*C. ariethium*	MeV	https://mev.tm4.org/#/about	Data processing and Metabolite annotation	[87]
	XLSAT software	https://www.xlstat.com/en/	Data processing	

**Table 3:** Statistical tools and databases used for metabolome data processing and annotation in legume biotic stress studies.

Legume	Statistical Tool/Database Name	Access Domain (URL, Accessed on 28 April 2022)	Function	Reference
*L. japonicus*	MapMan/PageMan	https://mapman.gabipd.org/mapman	Data processing Metabolite annotation	[108,109]
	MeV	https://mev.tm4.org/#/about	Data processing Metabolite annotation	
	Microsoft Excel	https://www.microsoft.com/en-za/	Data processing	
	MetaGeneAlyse	https://metagenealyse.mpimp-golm.mpg.de/	Data processing Metabolite annotation	
*L. corniculatus* *L. creticus* *L. tenius* *L. burttii* *L. uligino* *L. filicaulis*	GRaphPad (Prism)	https://www.graphpad.com/	Data processing	[110]
	MeV	https://mev.tm4.org/#/about	Data processing Metabolite annotation	
	MetaGeneAlyse	https://metagenealyse.mpimp-golm.mpg.de/	Data processing Metabolite annotation	
	Microsoft Excel	https://www.microsoft.com/en-za/	Data processing	
*Stylosanthes*	Microsoft Excel	https://www.microsoft.com/en-za/	Data processing	[26]
	SPSS	https://www.ibm.com/products/spss-statistics	Data processing	
	R	https://www.r-project.org/	Data processing	
	KEGG	https://www.genome.jp/kegg/kegg2.html	Metabolomic pathways	
*P. vulgaris*	MapMan	https://mapman.gabipd.org/mapman	Data processing and Metabolite annotation	[80]
	KEGG	https://www.genome.jp/kegg/kegg2.html	Metabolomic pathways	

## Data Availability

All the data are included in the main text.
